# THE IMPORTANCE OF CREATING SHARED EXPECTATIONS FOLLOWING NURSING HOME DISCHARGE

**DOI:** 10.1093/geroni/igz038.2575

**Published:** 2019-11-08

**Authors:** Whitney L Mills, Nathan Kerr, Kelsey Simons

**Affiliations:** 1 Center for Innovation in Long-Term Services and Supports, Providence VA Medical Center, Providence, Rhode Island, United States; 2 Uniformed Services University of the Health Sciences, North Bethesda, Maryland, United States; 3 VISN 2 Center of Excellence for Suicide Prevention, Canandaigua, New York, United States

## Abstract

Nursing home stays often represent a time of change for residents and their social networks (e.g., family, friends, and other social supports). Nursing home residents may be experiencing exacerbations of medical conditions, changes in physical functioning, and/or cognitive impairment. These changes often preclude the resident from returning to their pre-admission level of functioning, resulting in the need for adjustments for both residents and their social network. However, nursing home staff and residents/caregivers frequently have different expectations for post-discharge functioning. This may be the result of inadequate or unclear communication on the part of nursing home staff and/or residents and caregivers being unable or unwilling to internalize the information. The lack of shared expectations can leave residents and caregivers unprepared, creating unanticipated caregiving burdens for the social network and unwanted outcomes for the resident. We conducted interviews with 14 resident/caregiver dyads who experienced care transitions from VA nursing homes to examine expected and actual functioning and activities post-discharge. Using a qualitative content analysis approach, we identified several themes including marital strain related to mismatched expectations, impact of physical changes on Veterans’ social functioning, and differences between planned and actual post-discharge activities. We will discuss the importance of clear communication about expectations throughout the nursing home stay. We will also provide suggestions for improving discharge care planning to ensure creation of shared expectations and timely communication to allow residents and their social networks to adequately prepare for nursing home discharge.

